# Intracellular reduction in ATP levels contributes to CYT997‐induced suppression of metastasis of head and neck squamous carcinoma

**DOI:** 10.1111/jcmm.14017

**Published:** 2018-11-18

**Authors:** Xiangdong Zhao, Liwei Lang, Leilei He, Lixia Gao, David Chyan, Yuanping Xiong, Honglin Li, Hong Peng, Yong Teng

**Affiliations:** ^1^ Department of Otorhinolaryngology, Head and Neck Surgery Guangdong Second Provincial General Hospital Guangzhou Guangdong China; ^2^ Department of Oral Biology and Diagnostic Sciences Dental College of Georgia Augusta University Augusta Georgia; ^3^ Department of Biology College of Science and Mathematics Augusta University Augusta Georgia; ^4^ Department of Biochemistry and Molecular Biology Georgia Cancer Center Medical College of Georgia Augusta University Augusta Georgia; ^5^ Department of Medical Laboratory, Imaging and Radiologic Sciences College of Allied Health Augusta University Augusta Georgia

**Keywords:** ATP, CYT997, EMT, HNSCC, invasion and metastasis, mitochondria

## Abstract

The incidence rate of head and neck squamous cell carcinoma (HNSCC) has steadily increased over the past decade. However, treatment options for metastatic HNSCC are often limited and the 5‐year survival rate has remained static. Therefore, the development and assessment of more efficient but less toxic therapeutic strategies is an unmet need for treatment of more extensive HNSCC. Here, we report that CYT997, a novel microtubule‐disrupting agent, exerts strong activity in inhibiting HNSCC cell invasion and metastasis. The loss of invasion capacity by CYT997 was accompanied by an associated increase in cell adhesion and the reversal of epithelial‐mesenchymal transition (EMT). Increased expression of E‐cadherin protein and decreased expression of Vimentin protein became evident in HNSCC cells following CYT997 exposure, which were consistently observed in HNSCC xenografts from the mice receiving CYT997. Moreover, the capacity of invasive HNSCC cells to form pulmonary metastases was significantly blocked with CYT997 treatment, indicating that the diminishment of EMT traits contributes to CYT997‐suppressed metastasis. Intriguingly, CYT997 impaired intracellular ATP levels in HNSCC cells, at least in part, through its inhibitory effect on the mitochondrial protein IF1. The addition of ATP attenuated CYT997‐induced suppression of cell invasion, coupled with down‐regulation of E‐Cadherin and up‐regulation of Vimentin. These findings support a critical role of ATP levels in cell invasion and metastasis under the influence of CYT997. Collectively, our data unveil the mechanism involved in mediating CYT997 action, and provide preclinical rationale for possible clinical application of CYT997 as a novel therapeutic strategy against aggressive HNSCC.

## 1 INTRODUCTION

Head and neck squamous cell carcinoma (HNSCC) is the sixth most common malignancy worldwide, which is highly associated with alcohol, smoking, and HPV infection.^1^ Metastasis disease largely decreases the survival rate of patients with HNSCC and significantly affects treatment planning.^2^ Nevertheless, there remains much to be understood about the metastatic process and the therapeutic strategies against HNSCC metastasis. Generally, patients with early stage HNSCC, particularly those limited to the site of origin, are treated with either surgery or radiation therapy. If patients have more extensive cancers, they often receive chemotherapy. However, treatment options for recurrent and metastatic HNSCC are often limited, with palliative treatment generally offered in these situations. Therefore, the development and assessment of a new chemotherapy, which holds great potential for higher efficacy and lesser side effects, are imperative for the management of HNSCC, especially for metastatic tumours.

Microtubules are filamentous intracellular structures that are responsible for the maintenance of cell shape and the facilitation of cell motility.^3^ Targeting microtubules to disrupt their normal function within the cancer cell has proven to be one of the best classes of cancer chemotherapeutic drugs available in clinics to date.^4^ The microtubule‐targeting agents (MTAs), including Paclitaxel and Docetaxel, are very effective cancer drugs with therapeutic benefits in both hematopoietic and solid tumours. These drugs, as “stabilizers” or “destabilizers” of microtubules, potently suppress the dynamic stability of the microtubules to block mitotic progression and trigger apoptosis.^4^, ^5^ However, there is little evidence how MTAs affect cell motility, especially in the process of cancer cell invasion and metastasis.

CYT997, a new microtubule‐disrupting agent inhibiting tubulin polymerization and disrupting cellular microtubules, has been used as an anticancer chemo drug for a wide range of cancer types.^6^, ^7^, ^8^ The oral activity of CYT997 is more effective than most other MTAs. Phase I clinical trials have assessed the anticancer efficacy and safety of CYT997 in patients with solid tumours, and Phase II clinical trials are performing studies for the treatment of selected tumors.^8^, ^9^ We have found that CYT997 can trigger oxidative stress‐associated apoptosis and mTOR‐dependent autophagy in HNSCC cells, and CYT997‐induced autophagy appears to have a protective role against apoptosis by inhibiting the induction of excessively high reactive oxygen species (ROS).^10^ A superior inhibitory effect of CYT997 on prostate tumour growth and metastasis was demonstrated in our previous work, suggesting that CYT997 may also regulate the potential of invasion and metastasis of HNSCC cells.

Here, we study the anticancer activity of CYT997 in cell invasion and metastasis using cultured HNSCC cell lines and in vivo animal models. We show that CYT997 suppresses invasion and metastasis of HNSCC cells by increasing cell adhesion and reversing epithelial‐mesenchymal transition (EMT), which largely depends on the reduction in intracellular levels of ATP production. By testing the effects of the addition of ATP on CYT997‐induced suppression of invasion, we provide evidence that intracellular reduction in ATP levels has an important role in limiting HNSCC cell invasion/metastasis under the influence of CYT997.

## 2 MATERIALS AND METHODS

### 2.1 Cell lines and culture conditions

Invasive HN6 and HN12 cells derived from HNSCCs have been previously described.^11^, ^12^ All cells were maintained in Dulbecco's modified Eagle's medium containing 10% foetal bovine serum at 37°C in a humidified incubator supplied with 5% CO_2_.

### 2.2 Reagents, antibodies, and standard assays

CYT997 was purchased from Selleckchem (Houston, TX). Adenosine triphosphate (ATP) and β‐actin antibody were purchased from Sigma‐Aldrich (St Louis, MO). Antibodies that recognize HSP60, DRP1, PTPMT1, Cytochrome C, IF2, ATPAF1, IF1, and COXIV were obtained from Santa Cruz Biotechnology (Dallas, TX). EMT Antibody Sampler Kit and Tight Junction Antibody Sampler Kit were purchased from Cell Signaling Technology (Beverly, MA). Intracellular ATP levels were determined by ENLITEN^®^ ATP Assay System (Promega, Madison, MI). Western blot and wound healing assays were carried out as previously described.^6^, ^10^, ^13^, ^14^, ^15^

### 2.3 Cell viability and Matrigel invasion assays

Cell viability was determined by CellTiter‐Glo^®^ Luminescent cell viability assay (Promega, Madison, MI). For Matrigel invasion assays, cells were plated onto an 8‐mm invasion chamber covered with Matrigel (BD biosciences, San Jose, CA) at a density of 5 × 10^4^ cells/well and incubated for 24 hours. The chemotactic invasion of cells was induced by 10% FBS placed in the lower chamber. The invasive cells were fixed with 100% ethanol and then stained with 0.5% Crystal violet staining solution. Numbers of the stained invasive cells in nine randomly selected fields from triplicate chambers were counted in each experiment under an inverted phase‐contrast microscope.

### 2.4 Three‐dimensional (3D) tumour spheroid invasion assays

3D invasion assays were performed as we previously described.^16^ Briefly, 2 × 10^4^ cells were incubated overnight to form 3D spheroid in hanging droplet in a well of an inverted round bottom 96‐well plate, followed by adding150 μl mixture of Matrigel: DMEM without serum at the ratio 1:1. Then 150 μl complete culture medium containing double doses of CYT997 were added. After 4 days, invaded cells from spheroids were imaged under a microscope.

### 2.5 Mitochondrial staining

MitoTracker™ Red CMXRos obtained from Invitrogen (Carlsbad, CA) was used to label mitochondria within live cells utilizing the mitochondrial membrane potential according to manufacturer's instruction. The intensity of MitoTracker was measured by fluorescence microscope and flow cytometer. All flow cytometer data were analysed using FlowJo software (Tree Star, Ashland, OR).

### 2.6 Adhesion assays

The binding ability of cells to components of the extracellular matrix was determined by CytoSelect™ 48‐Well Cell Adhesion Assay (Cell Biolabs, San Diego, CA). Briefly, 1 × 10^5^ cells with or without CYT997 treatment were plated onto extracellular matrix‐coated wells and incubated for 90 minutes at 37°C under 5% CO_2_. Medium was removed, and cells were washed with PBS before the addition of Cell Stain Solution. The cells were then washed and dried, followed by the incubation of Extraction Solution for 10 minutes. The extracted samples were transferred to a 96‐well plate and the optical density measured at 560 nm.

### 2.7 Transmission electron microscopy (TEM)

Approximately 1.0 × 10^7^ cells were treated with 20 nmol/L CYT997 or DMSO for 24 hours and were harvested and fixed in 2% glutaraldehyde in 0.1 M sodium cacodylate (NaCAC) buffer (pH 7.4) for 45 minutes. The samples were postfixed in 2% osmium tetroxide in NaCAC, stained with 2% uranyl acetate, dehydrated with a graded ethanol series and embedded in Epon‐Araldite resin. Thin sections were cut with a Leica EM UC6 ultramicrotome (Leica Microsystems), collected on copper grids and stained with uranyl acetate and lead citrate. Cells were observed in a JEM 1230 transmission electron microscope and imaged with an UltraScan 4000 CCD camera and First Light Digital Camera Controller.

### 2.8 Animal studies

All animal experiments were approved by the Institutional Animal Care and Use Committee (IACUC) of Augusta University. Six‐week‐old NSG (NOD.Cg*‐Prkdc* ^*scid*^ *Il2rg* ^*tm1Wjl*^ */SzJ*) mice were purchased from the Jackson Laboratory (Bar Harbor, ME). To generate a xenotransplantation model, exponentially growing 1 × 10^6^ HN12 cells were suspended in 100 μl PBS and intravenously injected into tail vein of NSG mice. The tumour‐bearing mice were randomized (n = 5/group) for different treatment with vehicle and 20 mg/kg CYT997 daily by oral gavage respectively. The mice were sacrificed on treatment day 42, and the xenografts and lungs were removed and processed for Western blot and histopathological analysis.

### 2.9 Statistical analysis

Statistical analyses were performed by unpaired Student's *t* test for two group comparisons and ANOVA for multigroup comparisons at a significance level of *P *<* *0.05. Where indicated, the results were representative of at least three independent experiments performed in triplicate and were expressed as the mean ± SD.

## 3 RESULTS

### 3.1 CYT997 suppresses migration and invasion in HNSCC cells

**Figure 1:**
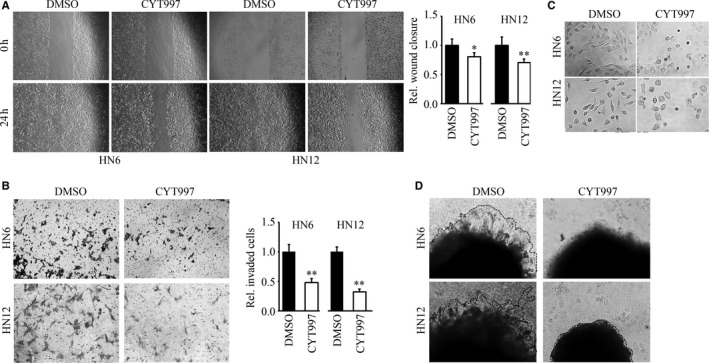
CYT997 inhibits cell migration and invasion of HNSCC cells. (A, B) The effect of CYT997 on cell migration and invasion. HN6 and HN12 cells were treated with 20 nmol/L CYT997 or DMSO for 24 h, and cell migration and invasion were determined by wound healing (A) and Matrigel invasion assays (B). Quantitative data from three independent experiments were shown in the right panel. C, The effect of CYT997 on cell shape. D, The effects of CYT997 on 3D invasion in Matrigel within 4 d. The extent of aggregated cells was determined within 20 min using a microscope. *P <* *0.05; **P <* *0.01

Our previous study has shown that CYT997 can inhibit cell viability and induce oxidative stress‐associated apoptosis in HNSCC cells.^10^ These data prompted us to investigate whether CYT997 has other anticancer potential in HNSCC cells. To mitigate the inhibitory effect on cell viability, we used 20 nmol/L CYT997 in the following in vitro studies, which was much lower than IC50 of 100 nmol/L in HNSCC cells. HN6 and HN12 are two highly invasive HNSCC cell lines, and no significant growth rate and cell death was noted in these cells, in the presence or absence of 20 nmol/L CYT997 (data not shown). However, CYT997 treatment led to a clear reduction in wound‐healing capability (Figure 1A). Matrigel invasion assays further showed reduced invasion potential in HN6 and HN12 cells following exposure of CYT997 (Figure 1B). To determine the contribution of EMT in CYT997‐induced suppression of cell invasion, we assessed the morphological changes of cells with or without CYT997 treatment. Long‐term treatment (3 days) of HN6 and HN12 cells with CYT997 reversed EMT as evidenced by converting from an elongated mesenchymal shape into a “cuboidal” epithelial structure (Figure 1C). Moreover, 3D tumour spheroid invasion followed over a period of 4 days as shown in Figure 1D revealed that invasion from HN6‐ and HN12‐derived spheroids was much less pronounced in CYT997 treatment compared with the control groups treated with DMSO.

### 3.2 CYT997 inhibits HNSCC cell EMT

**Figure 2:**
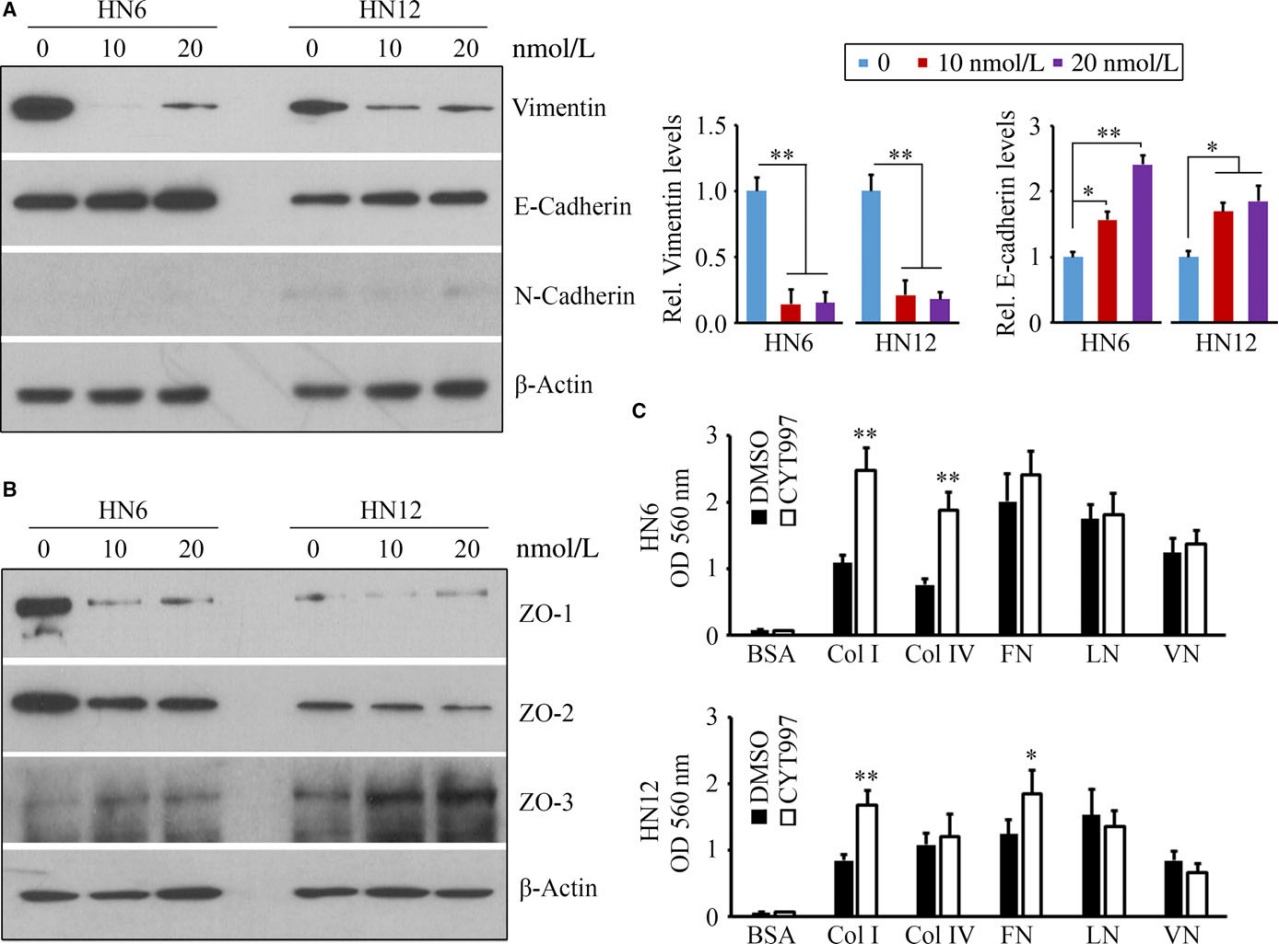
CYT997 inhibits HNSCC cell EMT. A, The effect of CYT997 on the levels of EMT‐related proteins. Representative Western blot data and quantitative data from three independent experiments were shown in the left and right panels, respectively. B, The effect of CYT997 on the levels of tight junction‐related proteins. C, The effect of CYT997 on cell adhesion. HN6 and HN12 cells were treated with 20 nmol/L CYT997 or DMSO for 24 h, and cell adhesion was determined by CytoSelect™ 48‐Well Cell Adhesion Assay Kit. *P <* *0.05; **P <* *0.01

To understand the mechanism underpinning CYT997‐induced suppression of invasion, we sought to determine the molecules mostly involved in EMT process. This investigation revealed a sharp decrease in protein levels of mesenchymal marker Vimentin following CYT997 treatment, which was accompanied by increased epithelial marker E‐Cadherin levels (Figure 2A). There were no significant changes in protein levels of other EMT‐related proteins, including N‐Cadherin, in the presence or absence of CYT997 (Figure 2A), excluding their functions in CYT997‐blocked EMT. These observations demonstrate that CYT997 diminishes EMT traits in mesenchymal‐like HNSCC cells through the regulation of Vimentin and E‐Cadherin expression levels. Given the fact that EMT is associated with the simultaneous repression of tight junction adhesion molecules,^17^ we then examined the levels of these proteins in cells with or without CYT997 treatment. The levels of ZO‐1 and ZO‐2 were decreased in HN6 cells but not HN12 cells (Figure 2B), whereas ZO‐3 was dramatically up‐regulated in both CYT997‐treated cells regardless of drug concentrations (Figure 2B). Nevertheless, CYT997 cannot induce significant changes in protein levels of other tight junction adhesion proteins, including Claudin‐1, CD2AP, and Afradin (data not shown). Additionally, cell adhesion assays showed an increased binding capacity of CYT997‐treated cells to collagen I compared with mock‐treated control cells (Figure 2C). In the presence of CYT997, adhesion to Collagen IV and fibronectin was enhanced in HN6 and HN12 respectively (Figure 2C). The analysis of cell adhesion to other extracellular matrix components revealed no difference in cell adhesion following CYT997 treatment (Figure 2C).

### 3.3 CYT997 suppresses head and neck tumour metastasis in mice

**Figure 3:**
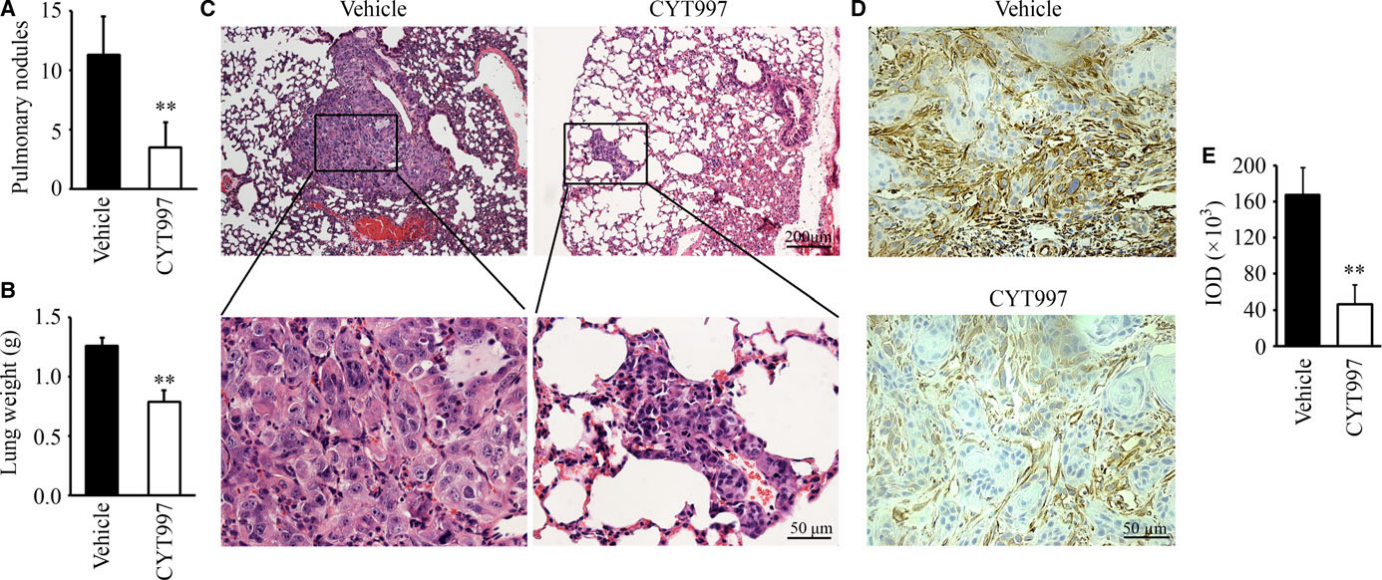
CYT997 suppresses head and neck tumour metastasis in mice. (A‐C) The effect of CYT997 on lung metastasis. The tumor‐bearing NSG mice were randomly divided into two groups (n = 5) for the indicated treatments, and were sacrificed on day 42 after treatment. The lungs were dissected and removed for histological analysis with H&E staining. The metastatic nodules on the pulmonary surface were counted (A) and the lungs were weighed (B). The lung sections were stained with H&E for pathological analysis (C). (D, E) The effect of CYT997 on the levels of Vimentin in the xenograft tumours. The representative IHC images were shown in (D) and quantification of IHC staining with Image pro‐Plus6.0 was shown in (E). *P <* *0.05; **P <* *0.01

Tail vein injection of mice with cancer cells is among the most used models for metastases, since some of the circulating tumour cells establish themselves in the mouse lung to create “experimental” metastatic foci. After injection of highly invasive HN12 cells into the tail vein of NSG mice, we used this model to determine the in vivo effects of CYT997 on metastatic potential. After 42 days of treatment, a reduced number and size of metastatic nodules on the lung surface were observed from mice receiving CYT997 compared with those receiving vehicle (sterile filtered phosphate‐buffered saline) (Figure 3A, B). Histopathological analysis further revealed that CYT997 treatment resulted in fewer and smaller tumour foci in the lung section compared with the control group (Figure 3C). Consistent with in vitro data, IHC analysis showed significantly reduced levels of Vimentin in tumour tissues treated by CYT997 (Figure 3D, E). These data indicate that CYT997 suppresses metastasis, at least in part, through inhibiting EMT of HNSCC cells.

### 3.4 CYT997 induces mitochondrial dysfunction and impairs intracellular ATP levels in HNSCC cells

**Figure 4:**
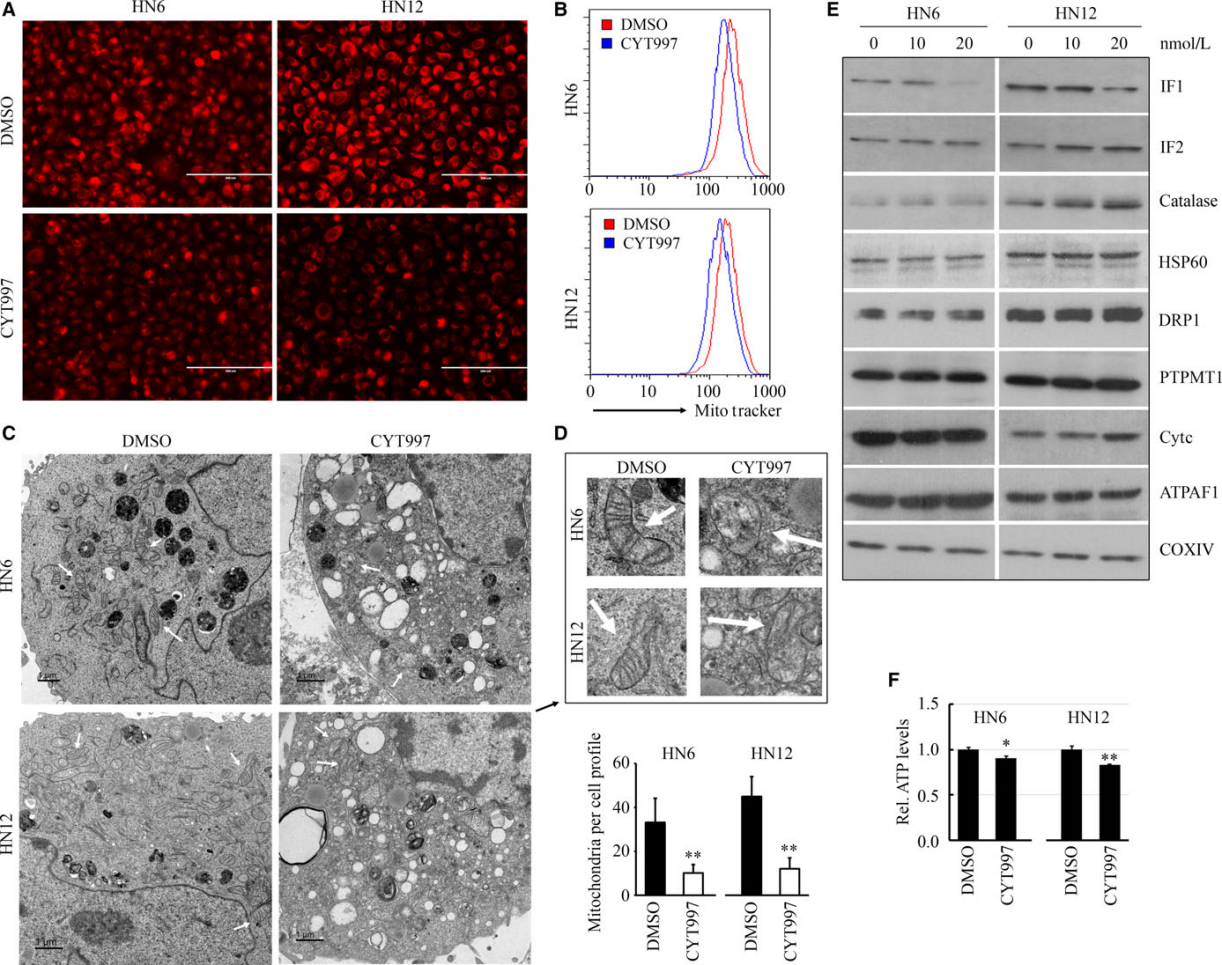
CYT997 induces mitochondrial dysfunction of HNSCC cells. (A, B) Fluorescent images and flow cytometer analysis of HN6 and HN12 cells loaded with MitoTracker™ Red CMXRos. C, TEM images of HN6 and HN12 cells in the presence or absence of 20 nmol/L CYT997. White arrows indicated mitochondria, and the number of normally shaped mitochondria was quantified from 20 randomly selected fields. D, Enlarged images of the mitochondria from (C). White arrows indicate the mitochondria in different treatments. E, The effect of CYT997 on the levels of main mitochondrial proteins. F, The effect of CYT997 on ATP release. HN6 and HN12 cells were treated with 20 nmol/L CYT997 or DMSO for 24 hours, and intracellular ATP was determined by ENLITEN ^®^ ATP Assay System. *P <* *0.05; **P <* *0.01

Our work has demonstrated that CYT997 can induce oxidative stress in HNSCC cells as evidenced by elevated ROS levels and enhanced superoxide release,^10^ raising the possibility that CYT997 may modulate mitochondrial function. To study the potential function of CYT997 in mitochondrial volume and shape changes, CYT997‐treated or nontreated HN6 and HN12 cells were stained with MitoTracker™ Red CMXRos, a red‐fluorescent dye that dependently accumulated upon mitochondrial membrane potential. The fluorescence signal from mitochondria was then determined by fluorescence microscope and flow cytometer. Both of these analyses showed that decreased levels of the fluorescence intensity in CYT997‐treated cells compared with the control cells (Figure 4A, B). TEM further revealed that CYT997 treatment led to significant changes in mitochondrial morphology, showing a high reduction in the number of normally shaped mitochondria with the lack of mitochondrial cristae (Figure 4C, D). We then sought to determine the levels of mitochondria‐localized proteins in the presence or absence of CYT997. Decreased protein levels of IF1 were observed when cells were treated with 20 nM CYT997 (Figure 4E). IF2 was only increased in HN12 cells following drug exposure, suggesting CYT997‐induced alterations in this protein is cell‐content dependent (Figure 4E). Other proteins examined in this study, including Catalase, HSP60, DRP1, PTPMT1, Cytochrome C, and ATPAF1, have not shown notable changes in their levels with or without CYT997 treatment (Figure 4E). Most of the ATP in cells is produced by ATP synthase in mitochondria, and IF1 is a natural inhibitor protein of mitochondrial ATP synthase.^18^ Given the fact that CTY997 induced repression of IF1 expression, we examined intracellular ATP levels by ENLITEN^®^ ATP Assay System. This analysis showed significantly lower levels in CYT997‐treated cells than in control cells (Figure 4F), indicating that CYT997 suppresses the production of ATP through inhibition of mitochondrial function in HNSCC cells.

### 3.5 Restoration of ATP attenuates CYT997‐induced suppression of invasion in HNSCC cells

**Figure 5:**
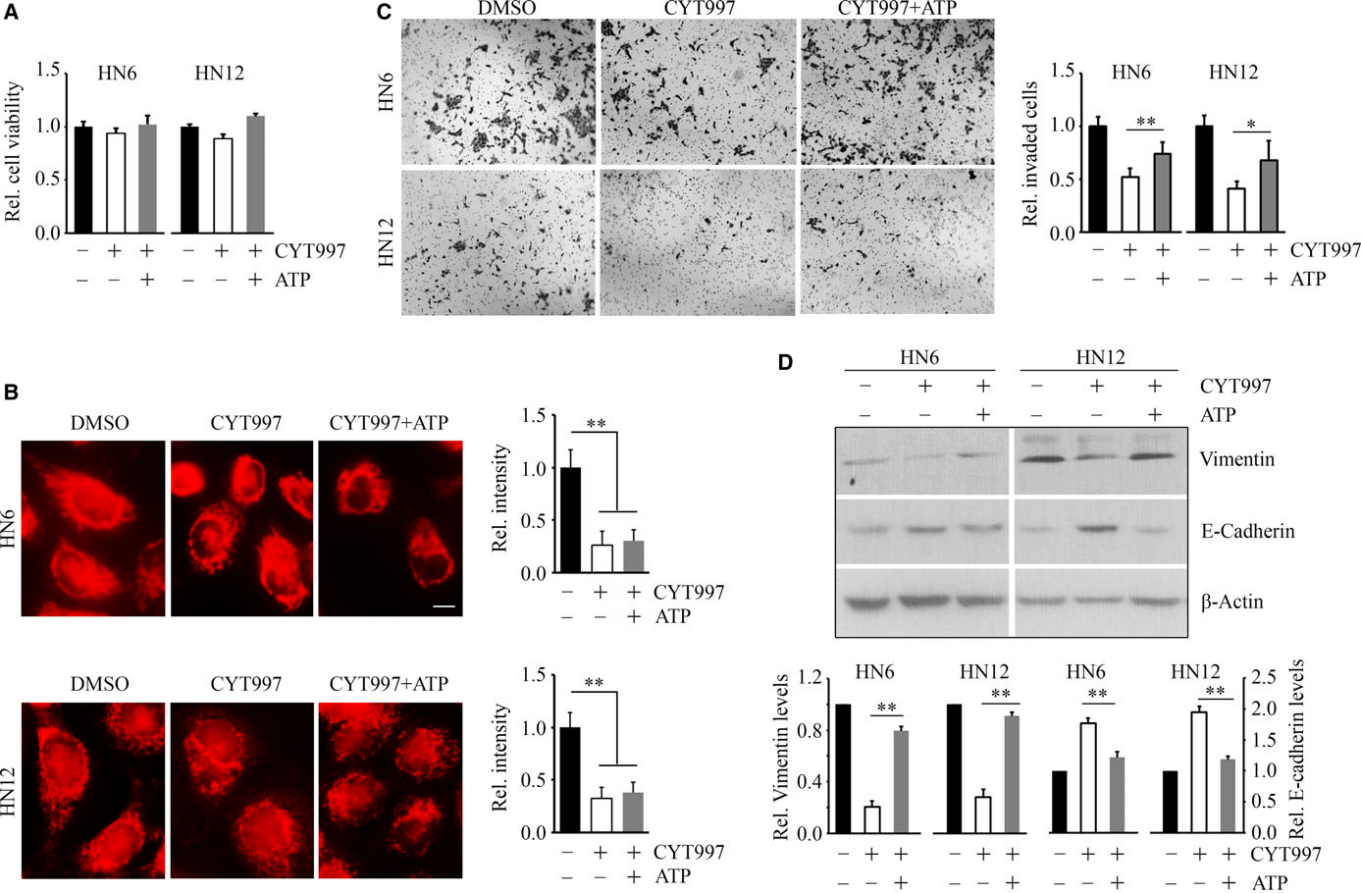
CYT997‐induced suppression of invasion can be rescued by the restoration of ATP in HNSCC cells. A, The effect of the addition of ATP on cell viability in the presence of CYT997. B, The effect of the addition of ATP on CYT997‐induced changes in mitochondrial morphology; scale bar = 10 μm. C, The effect of the addition of ATP on CYT997‐induced repression of cell invasion. Quantitative data from three independent experiments are shown in the right panel. D, The effect of the addition of ATP on the protein levels of Vimentin and E‐Cadherin in the presence or absence of CYT997. Quantitative data from three independent experiments were shown in the right panel. *P <* *0.05; **P <* *0.01

The maintenance of an adequate ATP supply is of crucial importance for the mechanisms of structural remodelling in cells with high shape plasticity.^19^ Therefore, we determined whether the loss of ATP played an essential role in CYT997‐induced suppression of cells invasion. CYT997 treatment alone appeared no effect on viability in both HN6 and HN12 cells, and addition of ATP to CYT997 did not change this phenotype (Figure 5A). MitoTracker staining showed the control cells displayed mitochondria that stained brightly and were a mixed reticulum with tubular and round form, whereas in the presence of CYT997, cells exhibited more round or fragmented mitochondria (Figure 5B). Addition of ATP did not rescue the changes in mitochondrial morphology induced by CYT997 (Figure 5B). However, increased invading cells were observed in both CYT997‐treated cells upon ATP administration (Figure 5C), indicating that invasion potential suppressed by CYT997 can be attenuated by ATP treatment. Moreover, under the influence of ATP, the protein levels of Vimentin and E‐Cadherin were almost returned to normal in CYT997‐treated cells as compared to the cells in absence of ATP (Figure 5D), suggesting that CYT997‐induced repression of ATP can reverse EMT phenotype in mesenchymal‐like HNSCC cells, resulting in decreased invasion and metastasis.

## 4 DISCUSSION

Current efficacy of chemotherapy in recurrent/metastatic HNSCC is far from satisfactory. Therefore, improving the effectiveness of chemotherapy as a core challenge is required for the clinical management of HNSCC. This study reports the anticancer activity of CYT997 in metastatic HNSCC cells, and demonstrates that CYT997 has potent effects on HNSCC cells not only by inhibiting cell proliferation and viability, but also by suppressing cell invasion and metastasis. The data present here suggest that CYT997 may be an effective treatment option for patients with HNSCC, especially for those with advanced‐stage tumours.

Oral drug administration is the most convenient method of medication and now more than 60% of marketed drugs are used as oral products. CYT997 can be well absorbed after oral administration, with mean Tmax and T1/2 of 1.1 and 3.2 hours, respectively.^9^ In our previous animal study, tumour‐bearing mice treated with CYT997 by oral gavage showed significantly smaller xenografts compared with control mice.^10^ We have not successfully identified metastatic HNSCC cells in the lungs of mice in the previous study, which may be due to the termination of the animal experiment too early. In this study, we inoculated a low number of HN12 cells (5‐fold cells less than the last experiment) into the flank of NSG mice and extended the experimental window for three more weeks, allowing tumour cells to develop metastatic tumours before the tumour burden became excessive. It is clear that, in the absence of CYT997, highly invasive HN12 cells can metastasize to the lung from a primary site in NSG mice. In contrast, much fewer cancer cells can be observed in the lungs of the mice following CYT997 treatment, indicating that CYT997 has a superior anticancer activity by inhibiting tumour growth and metastasis, coincidently. Recently, an orthotopic mouse model of HNSCC has been established by the implantation of cancer cells into the floor of a mouse's mouth, allowing us to better study the anticancer effects of CYT997. Pharmacokinetics and pharmacodynamics of this drug will be also beneficial from using an orthotopic mouse model, which paves the way to the successful, practical use of CYT997 in cancer treatment.

**Figure 6:**
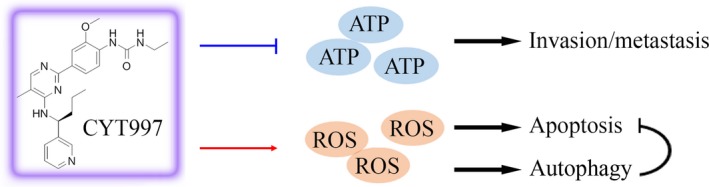
Schematic representation of drug mechanisms of CYT997 in HNSCC cells. In HNSCC cells, induction of ROS by CYT997 triggers autophagy to attenuate its mediated apoptosis, and blockade of ATP release by CYT997 suppresses cell invasion and metastasis

CYT997 is reported as an available antiangiogenic agent to suppress the local invasion and distant metastasis of malignant tumours, but whether CYT997 plays an importance in inhibition of EMT in cancer cells remains unclear. Here we reveal that CYT997 can induce significant repression of invasion and EMT phenotypes, which is abrogated by ATP treatment. It appears that CYT997 decreases ATP production and, in turn, up‐regulates E‐Cadherin and down‐regulates Vimentin to facilitate the reversal of EMT, resulting in the decreased invasion and metastasis of HNSCC cells. Elevated ROS generation is engaged in CYT997‐induced inhibition of cell viability.^10^ We treated HN6 and HN12 cells with NAC, an antioxidant with the ability to minimize oxidative stress, in the presence or absence of CYT997. No significant changes in cell invasion and EMT were seen with or without NAC treatment (data not shown), excluding the role of ROS in cell movement‐related phenotype in CYT997 treatment. It looks that, on one hand, CYT997‐induced ROS is the key player involved in the enhancement of apoptosis and autophagy (Figure 6), and increased autophagy by CYT997 plays a prosurvival role through inhibiting ROS‐dependent apoptosis in HNSCC cells.^10^ On the other hand, CYT997‐induced blockade of ATP release suppresses HNSCC cell invasion and metastasis (Figure 6). These findings raise concerns that CYT997 may be applied in the execution of new chemotherapeutics for HNSCC treatment.

Clear resolution between apoptotic and non‐apoptotic cells can be observed with MitoTracker™ Red dye CMXRos. Our study reveals that 20 nmol/L CYT997 is not sufficient to induce apoptosis of HNSCC cells, while at this concentration, CYT997‐treated HNSCC cells exhibited decreased CMXRos fluorescence compared with the control cells. This observation suggests that CYT997 at low dosage plays a role in reducing the mitochondrial mass of HNSCC cells, rather than in inducing apoptosis. We have demonstrated that CYT997 can alter mitochondrial morphology in HNSCC cells. It is possible that distribution of mitochondria may also affected upon CYT997 treatment. However, distinguishing this from cell shape changes induced by CYT997 is still a challenge in this study.

As the “principal energy currency,” ATP is involved in metabolism and most of the other cellular activities.^19^ However, the role of intracellular ATP in sustaining cell motility is very obscure, which may depend on its levels and distribution pattern. Mitochondria is the site of ATP generation, and the dysfunction of mitochondria in HNSCC cells has been found in the presence of CYT997, which may explain how the cellular levels of ATP production were impaired. Tumour microenvironment is ATP rich, suggesting a role for purinergic signalling in cancer development and progression. Interestingly, addition of ATP in CYT997 treatment can attenuates CYT997‐induced suppression of HNSCC cell invasion (Figure 5), supporting a critical role of ATP levels in chemo drug treatment. However, the underlying molecular mechanisms are still unclear, which will be defined in our future study. Analysis of mitochondrial protein expression profile shows a marked decrease in IF1 protein levels in the presence of CYT997. IF1 is an endogenous inhibitor protein of mitochondrial ATP synthase. Suppression of IF1 expression in human cell line HL‐1, C2C12, and HeLa reduced cellular ATP levels coupled with increased ROS (reactive oxygen species),^20^, ^21^ indicating that IF1 plays a critical role in prevention of wasteful ATP consumption and in the suppression of ROS generation. Our data imply that IF1 may contribute to CYT997‐induced suppression of cell invasion and metastasis via the regulation of cellular ATP levels. One of the follow‐up studies is to determine whether its function is associated with the extreme mitochondrial morphological changes and reduced CMXRos staining.

ZO‐1, ZO‐2, and ZO‐3 are a family of tight junction associated proteins that function as cross linkers, anchoring the tight junction strand proteins to the actin‐based cytoskeleton. Surprisingly, treatment with sub‐lethal dose of CYT997 only leads to increased ZO‐3 protein levels. Although mice lacking TJP3/ZO‐3 show no apparent phenotype,^22^ it is critical for barrier function of the enveloping cell layer and osmoregulation in early stages of zebrafish development.^23^ How ZO‐3 is up‐regulated by CYT997 is one of our follow‐up investigations. In summary, we demonstrate that CYT997 strongly suppresses invasion and metastasis of HNSCC cells, which is accompanied by reduction in intracellular ATP production. The addition of ATP levels hampers the efficacy of CYT997 in inhibition of cancer invasion and metastasis, which suggests that blockage of ATP by CYT997 is one of the main mechanisms in understanding drug‐induced changes in cancer cell motility. Our findings provide a rationale to use CYT997 as an effective therapeutic strategy for either primary or metastatic HNSCC.

## CONFLICTS OF INTEREST

The authors declare no conflict of interest.
